# Vulnerable to heat stress: gaps in international standard metric thresholds

**DOI:** 10.1007/s00484-024-02783-6

**Published:** 2024-09-20

**Authors:** C. Brimicombe, C. Gao, I. M. Otto

**Affiliations:** 1 Wegener Centre for Climate and Global Change, University of Graz, Brandhofgasse 5, Graz, 8010 Austria; 2 Aerosol and Climate Lab, Division of Ergonomics and Aerosol Technology, Department of Design Sciences, Faculty of Engineering (LTH), Lund University, Lund, Sweden

**Keywords:** Occupational standards, Thermal comfort, Extreme heat, Workforce, Climate change

## Abstract

Exposure time to heat is increasing with climate change. Heat exposure thresholds are important to inform heat early warning systems, and legislation and guidance for safety in the workplace. It has already been stated that thresholds can be lower for vulnerable groups, including the elderly, pregnant women, children, and those with pre-existing medical conditions due to their reduced ability to thermoregulate their temperature or apply cooling strategies. However, the Wet Bulb Globe Temperature (WBGT) proposed by the international standard organisation (ISO 7243:2017), only takes into account thresholds based on acclimatization status. Therefore in this study we carried out a PRISMA systematic keyword search of “Wet Bulb Globe Temperature” of the Scopus abstract and citation database in August 2023 and a meta-analysis of text extracted from the identified 913 international studies published between December 1957 and July 2023, to investigate heat stress thresholds for different population groups. We find that different thresholds are considered as an indication of heat stress for different population groups. However, critical gaps were identified for the most vulnerable populations, and there are lower numbers of studies on women. Most studies researched adult populations between the ages of 18 and 55 (*n* = 491), failing to include the youngest and oldest members of society. Based on these findings, we call for targeted investigations to inform effective heat action policies and set early warning thresholds to ensure the safety and wellbeing of the entire population.

**Supplementary Information:**

The online version contains supplementary material available at 10.1007/s00484-024-02783-6.

## Introduction

Heat stress is a growing risk for people around the world (IPCC et al. 2021). Extreme heat is an important aspect of global warming and our changing climate. Evidence shows that heat exposure thresholds and survivability limits could be breached soon in some regions and in a growing number of countries (Kenney et al. 2022). Nearly one in three heatwave deaths can be attributed to climate change (Vicedo-Cabrera et al. 2021). In addition, many regions have predicted productivity and economic losses due to the people’s increased exposure to extreme heat (Simpson et al. 2021). Such extreme heat puts pressure on cultural working practices and livelihoods (Hitchings 2022).

Heat stress is defined as “*the build-up of body heat generated either internally by muscle use or externally by the environment”* (McGregor and Vanos 2018) Researchers have established many heat stress indicators to indicate the risk of heat (Ioannou et al. 2022). Heat stress can be measured and evaluated by the international standard Wet Bulb Globe Temperature (WBGT) (Parsons, 2006), a standard which was originally developed by the U.S. Navy in the 1950s to indicate safe heat levels for training in the military (Minard 1961). Three components that influence the body’s response to the thermal environment make up the WBGT: the wet bulb temperature (i.e. an indication of the air moisture level, and indirect indication of air velocity), air temperature (i.e. how hot the air is), and globe temperature (i.e. the incidence of radiation on the body) (Minard 1961). The WBGT (ISO 7243:2017) is generally used to outline safe heat thresholds for workers in indoor and outdoor settings, with bespoke analyses created and guidelines made for working and rest regimes (Jacklitsch et al. 2016). A wide range of occupational sectors use the WBGT, such as those employing construction workers, professional football players, and foundry workers (Kjellstrom et al. 2009).

The WBGT outperforms all other available heat and thermal comfort indices in terms of screening heat stress(Ioannou et al. 2022). However, people are being exposed to steadily increasing amounts of heat stress, and there are different limits to people’s abilities to work and survive (Kenney et al. 2022). These differences arise from an individual’s vulnerability to heat, type of clothing, health and acclimatisation status, as well as whether the person is familiar with the surrounding thermal environment (Nazarian et al. 2019). In our study, we refer to the following definition of vulnerability: *“The conditions determined by physical*, * social*, * economic and environmental factors or processes which increase the susceptibility of an individual*, * a community*, * assets or systems to the impacts of hazards”* (Nations Office for Disaster Risk Reduction, 2015).

People who are the most vulnerable to heat have a limited ability to thermoregulate, meaning that their body responds less efficiently to the thermal environment and biologically cools down or heats up more quickly (Kuht and Farmery 2021). In addition, groups can be vulnerable due to their limited adaptive capacity, for example due to insufficient access to clean water, which can lead to dehydration during periods of extreme heat (Xu et al. 2012). Broadly, groups defined as vulnerable include the elderly (people over 65 years of age), children (people under 12 years of age), people with pre-existing medical conditions (i.e. heart or lung diseases), pregnant women, and outdoor workers (Otto et al. 2017). Researchers must consider the varying responses of vulnerable groups to heat to assess whether the heat stress thresholds as defined by the WBGT adequately account for these differences because WBGT is the international occupational standard metric.

In this study, we used a reproducible systematic review framework to identify relevant texts and conducted a meta-analysis of text extracted from published articles abstracts on WBGT by using the PRISMA framework (Page et al. 2021). First, we identified relevant studies on heat stress published between December 1957 and July 2023 by searching the Scopus database using specific keywords. Second, we explored measures of WBGT and defined thresholds in the 913 identified studies to determine whether the evidence base already exists for different thresholds between population groups. We asked three questions: (1) *What is the distribution of WBGT across geographies and occupations?* (2) *How do studies consider age and sex?* (3) *How do the thresholds of workability differ in different studies?*

Below, we first outline the methods used and then the overall results before categorising the results by age, sex, and occupational or physiological studies. Then, we present the results of the meta-analysis conducted to determine thresholds before discussing the results in the context of the broader body of literature. Key themes addressed in this discussion are main gaps that still exist, the many methods used to approximate the WBGT, and strategies that can be employed to bridge emerging gaps. Finally, we highlight where gaps remain in our understanding of populations vulnerable to heat to create a focal point for future research.

## Methods

We conducted a systematic review of the literature on the wet bulb globe temperature (WBGT). We were guided by the Preferred Reporting Items for Systematic Reviews and Meta-Analyses (PRISMA) checklist (Page et al. 2021). The full methodology was registered on OSF Registries and is available at: https://osf.io/g43sb, and the checklist of items and the Microsoft Excel spreadsheet of all extractions is provided in the supplementary material.

### Search

This search was carried out in August 2023 using Scopus, one of the most comprehensive literature databases available. We searched for studies that mentioned the WBGT by searching for the phrase “Wet Bulb Globe Temperature” in abstracts and keywords. This search returned *N* = 1128 hits. We also investigated searching using the terms “heat stress” and “WBGT” but further exploration showed many duplicate studies and many studies that were not relevant in our context.

### Inclusion criteria

When conducting this search, we only included human-focused research that was carried out to evaluate heat stress through the measurement of the WBGT. The following inclusion criteria were applied: peer-reviewed papers available in the English language and that were published in the period of December 1957 to July 2023. Grey literature was excluded. All study designs were considered eligible, except for systematic reviews, qualitative studies, and investigations that employed modelling to explore potential or hypothetical associations between heat exposure and health outcomes. Applying this criterion resulted in the identification of *n* = 913 relevant studies.

### Screening and selection

**Fig. 1:**
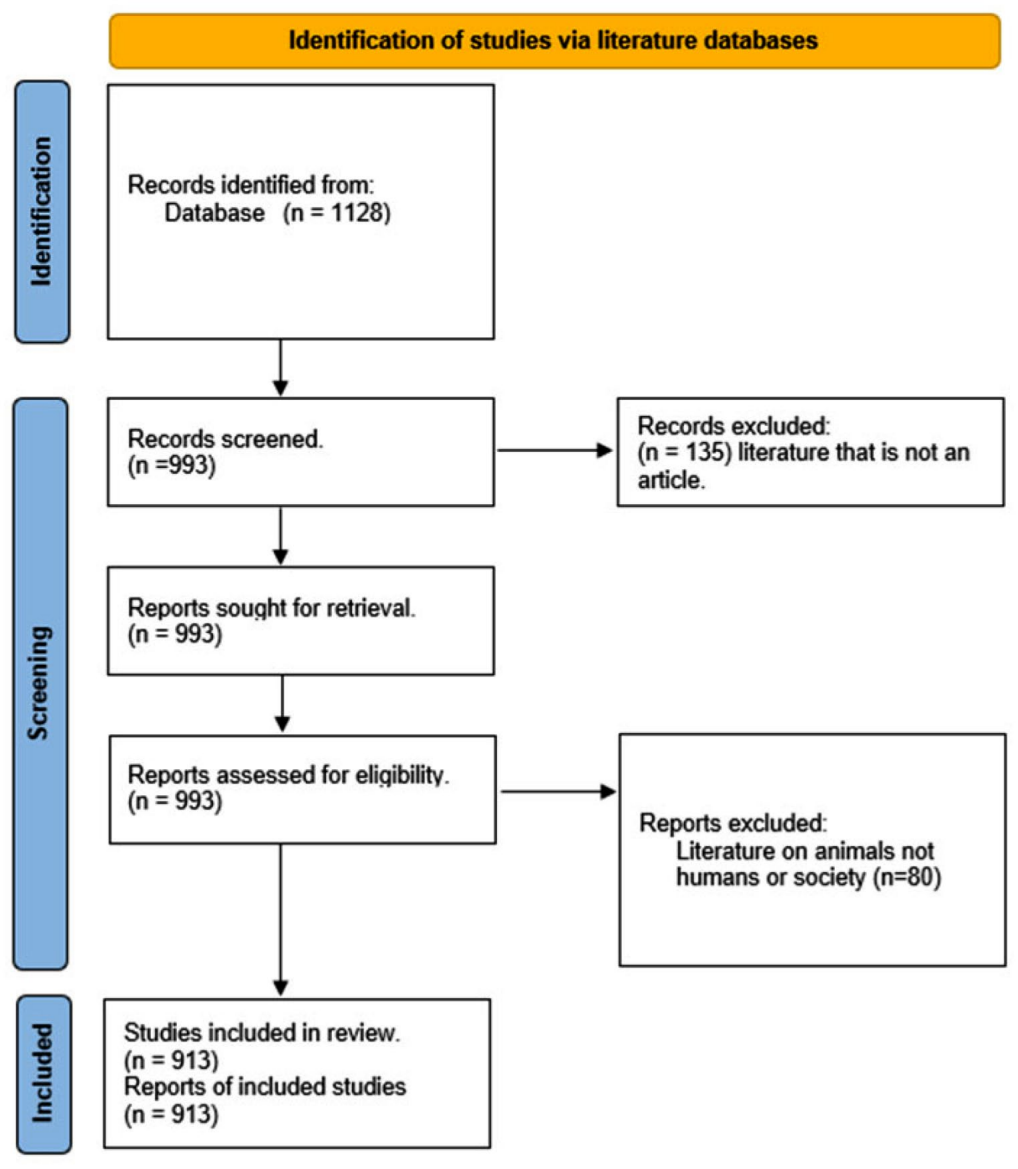
PRISMA flowchart illustrating systematic search, selection, and extraction process

We imported all references retrieved into Microsoft Excel spreadsheet editor program. One researcher (CB) screened the titles and abstracts of all records and checked whether their inclusion was appropriate by conferring with the other authors (CG and IMO). However, no further criterion ranking was conducted to include the broadest range of studies. The PRISMA flow chart illustrates how documents were screened and selection decisions were made (Fig. 1).

### Data extraction

A separate Microsoft Excel spreadsheet was then created and used to extract key information relevant to the study objectives from all included references. Key datapoints extracted from this dataset included the year of publication, evidence of acclimatisation and protective clothing use, country where the study was carried out, field of study, type of WBGT, age and sex of sample population members. One author (CB) extracted these datapoints, and these data were presented to other authors for consensus.

### Synthesis of results

Considering all the literature found within our inclusion criteria, we generated a variety of plots to visually represent how age groups and sex, were represented in the studies. This step was taken to identify knowledge gaps and vulnerable populations in the literature. For each demographic category, we precisely defined thematic criteria based on the text extracted from the references (i.e. if a study indicated that the sample population consisted of children, this study was included in the child category).

We carried out a meta-analysis of the data and consolidated the various thresholds cited in the studies denoting levels of heat stress, focusing exclusively on the outdoor WBGT metric. This meta-analysis excluded studies on alternative metrics, such as the indoor WBGT. The categories utilised were derived from the international standard documentation shown in Table 1 (Int Org Standard 2017). We evaluated and depicted this information based on sex, age, acclimatisation level and protective clothing. All analytical procedures were carried out by using the Python programming language.

**Table 1:** Heat stress thresholds for WBGT effective from the International Occupational standards Work for both acclimatised and un-acclimatised people

Metabolic rate(class)	WBGT reference limit persons acclimatised to heat (°C)	WBGT reference persons unacclimated to heat (°C)
Resting metabolic rate (115 W)	33	32
Low metabolic rate (180 W)	30	29
Moderate metabolic rate (300 W)	28	26
High metabolic rate (415 W)	26	23
Very High metabolic rate (520)	25	20

## Results

**Fig. 2:**
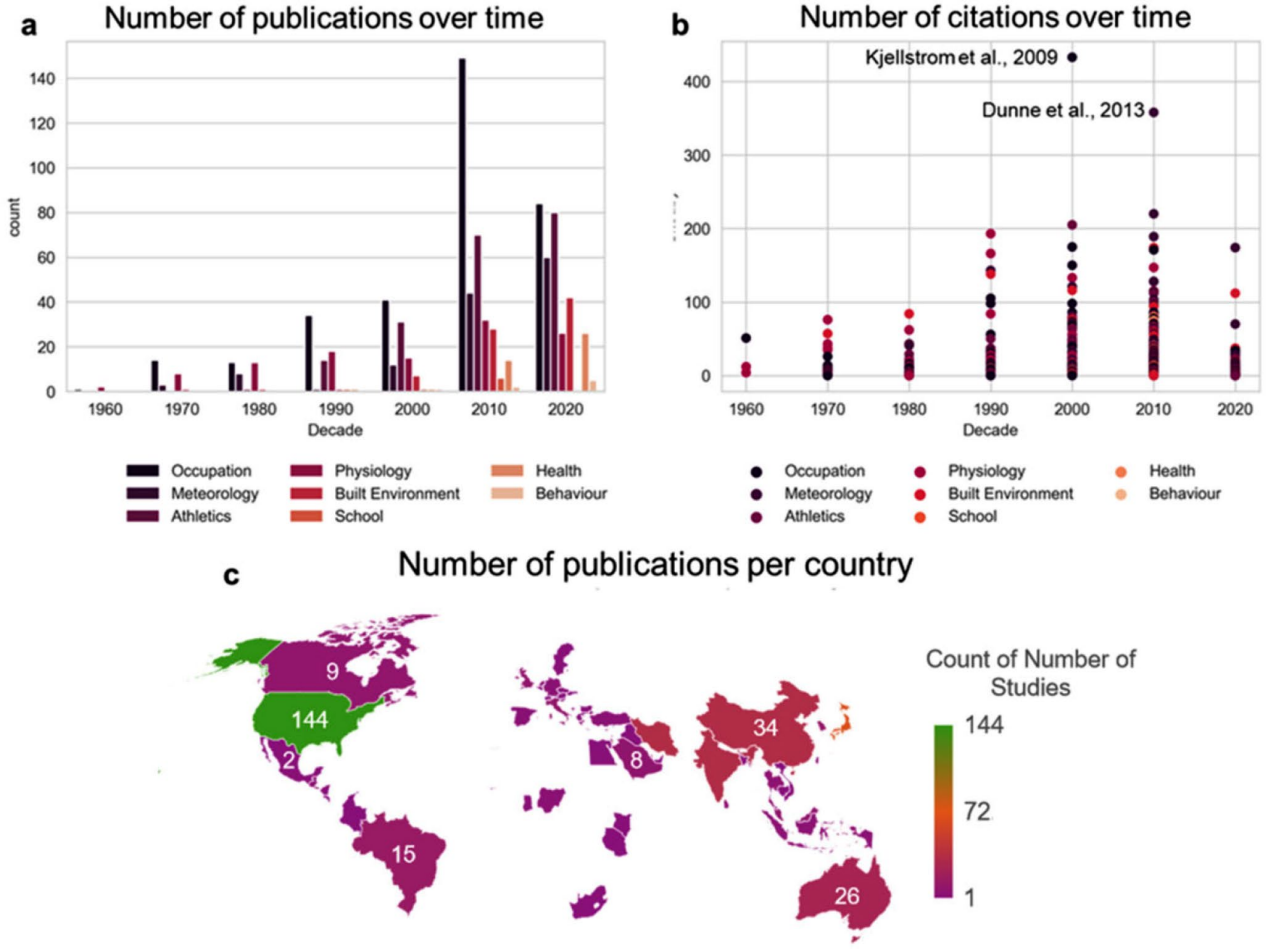
The distribution of studies by occupational field and geographical region for the WBGT. **A**) The number of studies per field, grouped by the decade in which they were published. **B**)The citations per study per field, grouped by the decade in which they were published. **C**) The number of studies that took place in each country (*N* = 513), where the study took place from December 1957 until July 2023

The body of WBGT literature has grown over time since the first study was published in December 1957. Most studies are clustered around types of occupation with over 140 studies being published between 2010 and 2019. In addition, the studies described in these papers are widely distributed in terms of occupations and geographical regions covered (Fig. 2). The occupation category contains the most studies overall (*n* = 336), while relatively few studies have been published on behaviour (*n* = 8) and schools and neonatal care units (*n* = 8) (Fig. 1a). Within the physiological studies there was consistency in the methods being used, with most studies taking place in a lab setting. Within the occupational studies there was a mix in qualitive studies and quantitative studies, with most being based on a survey type design.

The category of meteorology contains the 2nd most frequently cited paper, namely that by Dunne et al. (2013) (*n* = 358), even though this category contains fewer studies overall than the category of athletics another large cluster from our analysis. The most frequently cited paper is on workplace heat stress, health and productivity by Kjellstrom et al. (2009) (cited by *n* = 433) (Fig. 2b). There is relatively good coverage of the use of the WBGT by country (*n = 513)*, demonstrating that it is widely applied as an international standard. Nevertheless, noticeable disparities exist, and particularly in Sub-Saharan Africa and parts of Southern America, which are historically recognised as regions highly susceptible to heat (Feron et al. 2019; Russo et al. 2017). Additionally, our analysis reveals knowledge gaps for the region of Eastern Europe, with studies only for Slovenia and Slovakia, which is known to export significant amounts of agricultural products and to employ a substantial number of outdoor workers (Swinnen et al. 2017).

**Fig. 3:**
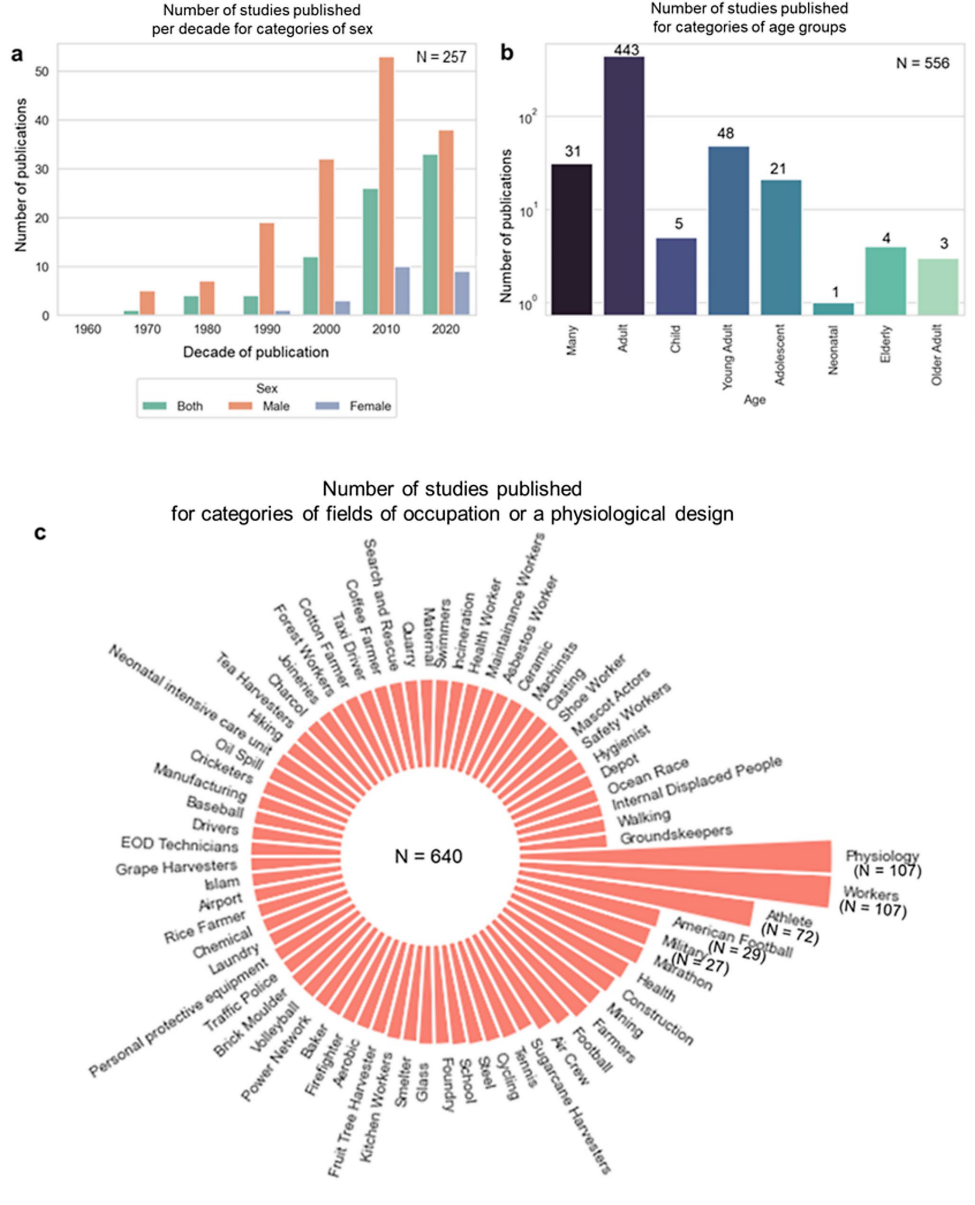
The number of studies grouped into different categories based on heat vulnerability. **a**) Number of studies published each decade, focusing on different sex groups. **b**) Number of studies published each decade, focusing on different age groups, with multiple age categories, defined by years of age – many (mixed age groups), adult (25 to 55), young adult (18 to 25), child (1 to 12), adolescent (12 to 18), older adult (55 to 65), elderly (65+), neonatal: up to 1 month. **c**) Field of occupations considered in the studies; 640 studies are occupational or studies of a physiological design

In total, authors of 567 studies examined some type of outdoor WBGT, but gaps were recognised for heat-vulnerable groups. Where the effects of different sex were explored (*n* = 257), there is an unbalance within studies with most clustering on males (*n* = 154/257) (Fig. 3a). In the studies examining the influence of heat on sex, three studied pregnant women (Bonell et al. 2020, 2023; Nainggolan et al. 2021), and only one study assessed other aspects of female health (Lei et al. 2017). We also observed an age bias in studies where the effects of age were considered (*n* = 556), as most studies were conducted on adults (*n* = 443) of 25 to 55 years of age and younger adults of 18 to 25 years of age (*n* = 48) (Fig. 3b). No studies were carried out on infants of one month to one year in age. Very few studies were carried out on the vulnerable groups of neonates up to 28 days old (*n* = 1), children of one year to 5 years (*n* = 5), older adults of 55 to 65 years (*n* = 3) and those above 65 years of age (*n* = 4). Only two studies conducted on people older than 65 explicitly mentioned their sex (Kajii and Kawashima 1993; Kwon et al. 2015). No studies placed an exclusive focus on elderly and/or older females, although women have a longer life expectancy than men (Barford et al. 2006).

Most studies categorised as occupational or physiological studies (Fig. 3c) were carried out on different types of workers, were purely physiology studies (*n* = 107), or examined types of athletic sports (including rugby and sumo wrestling) (*n* = 72). In physiological lab studies there was consistency in types of methodologies being used. Some notable outdoor industries with few studies are aquaculture (Vannuccini et al. 2018), with a reported 200 million direct or indirect workers worldwide, and waste collection, with about 20 million workers (Yang et al. 2018). Other industries with few studies are those employing emergency service providers, traffic police, and firefighters. In addition, very few studies considered pre-existing medical conditions in the study populations.

In 253 studies, the WBGT metric was not measured and was measured by using approximations such as the environmental stress index (ESI) or Swedish WBGT. In addition, sometimes WBGT was measured by globe temperature or wet bulb temperature, which are components of WBGT. Our understanding of thresholds for Outdoor WBGT comes mostly from Athletic studies (*n* = 97/207) and Occupational studies (*n* = 69/207), only 10 meteorological studies list thresholds. Across Outdoor WBGT studies considering thresholds there is a high variance amongst studies (25.1), showing a large spread across thresholds.

**Fig. 4:**
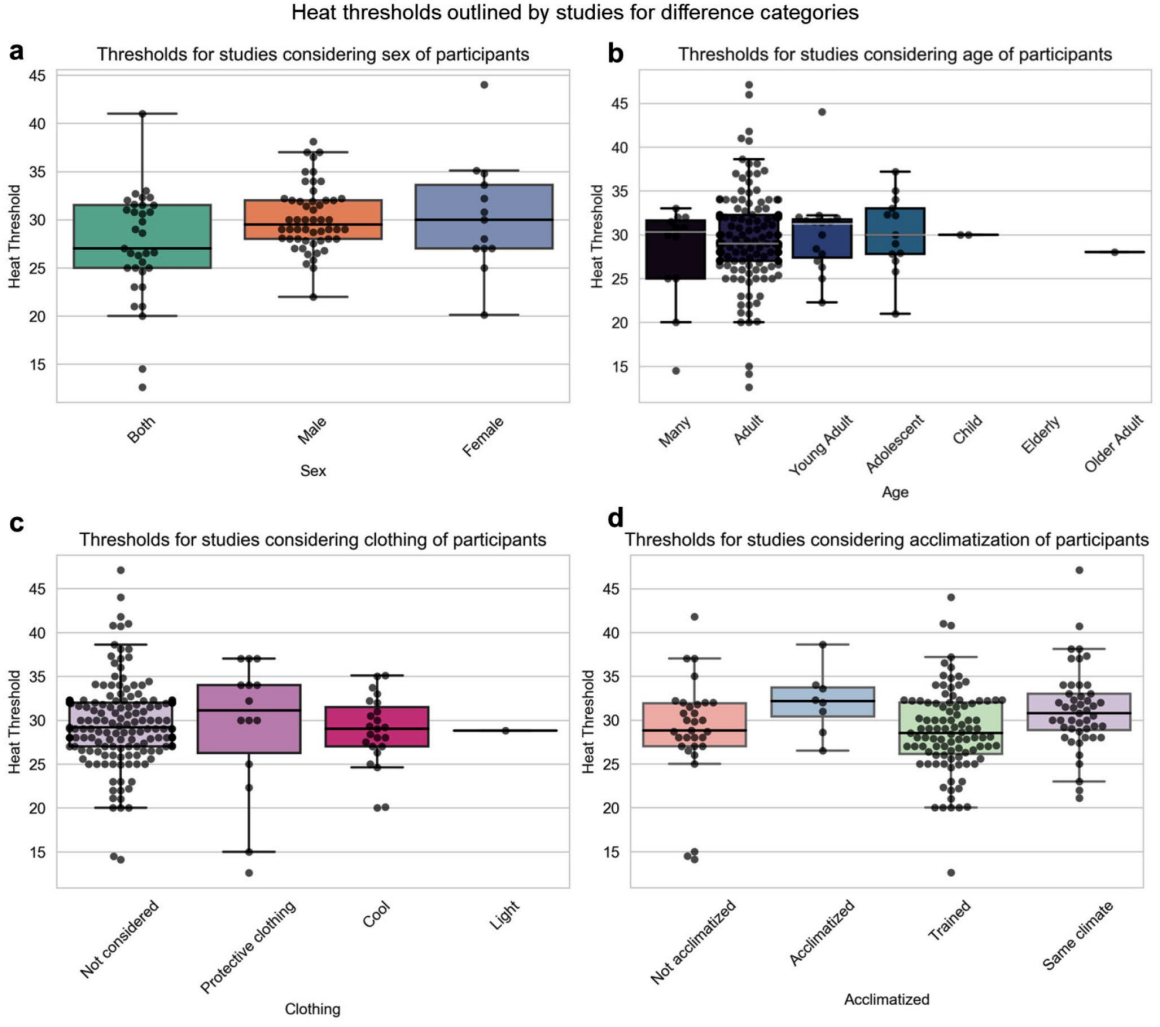
The thresholds for heat stress used in studies on the outdoor WBGT for different categories, namely threshold groups by **a**) sex, **b**) age, focusing on different age groups, with multiple age categories, defined by years of age – many (mixed age groups), adult (25 to 55), young adult (18 to 25), child (1 to 12), adolescent (12 to 18), older adult (55 to 65), elderly (65+), (c) type of clothing, and (d) level of acclimatisation

Outdoor WBGT thresholds used to define the likelihood that heat stress will occur differed according to sex, age, acclimatisation, and type of clothing (Fig. 4). Some studies examined different threshold ranges as indications of the heat stress risk for the sex of an individual, conclusions consistent with the differences in the studies (Beshir and Ramsey 1981; Green et al. 2000). A median WBGT threshold of 30.3 °C is cited for both sexes, 30.0 °C for males, and 30.8 °C for females (Fig. 4a). In studies where researchers examined the effects of protective clothing a higher median threshold of around 33 °C was found, whereas an absence of such clothing indicated a median threshold of around 29 °C (Fig. 4c).

When the effects of age were examined, we observed that the studies also report different threshold ranges as indications of heat stress risk (Fig. 4b). Some studies suggest that these thresholds can significantly change, depending on a person’s age (Oka et al. 2023; Saha et al. 2008). The median WBGT threshold is 29.5 °C for adolescents, 30.0 °C for adults, children, and older adults, and 31.5 °C for young adults (Fig. 4b), indicating work of a low metabolic rate for both acclimatised and unacclimatised people (Table 1) (Int Org Standard 2017). The median threshold for acclimatised individuals, i.e. where the study population is from the same climate and is comfortable with the thermal environment, is higher than for individuals who are not acclimatised (Fig. 4d). In all cases, the studies included a range of values higher than the maximum WBGT threshold of 33 °C for the resting metabolic rate of acclimatized people (Int Org Standard 2017).

## Discussion

### Key gaps emerge

Our results provide sufficient evidence to suggest that the thresholds outlined in international guidance could be adjusted to align with those presented in the literature for various population groups. However, we observed a lack of studies on older adults and the elderly across all themes of study, although these groups represent nearly 10% of the global population. The fact that the global population is ageing is apparent in many parts of the world (Kulik et al. 2014). In addition, no studies were identified as having been conducted on infants, and very few studies, on children and neonates. Some studies state that infants and children are more vulnerable to heat due to their smaller size, as their bodies heat up more quickly (Rowland 2008; Tsuzuki-Hayakawa et al. 1995). In addition, children are reported as being less able to take action to cool their bodies, because they often depend on an adult to provide them with water or help them dress appropriately (Xu et al. 2012). Our results indicate that it is important to collect evidence for these vulnerable groups to be able to identify heat thresholds for these group and design targeted policies for reducing the risk of heat. Our findings also showed that studies should use a similar design to studies that have been carried out for other age groups, placing a focus on whether the WBGT and the defined thresholds are appropriate for children, infants, or neonates by conducting effective temperature measurements and, where possible, using qualitative indicators such as subjective wellbeing. In addition, studies should carefully outline ethical considerations for all population groups, as this will help other researchers to conduct safer studies with research participants.

A gap remains around heat safety thresholds for females, with only 40% of studies for WBGT considering the female sex, with 9% only focusing on women (Fig. 2a). We consider this a bias in the overall literature base on WBGT. However, in part, this is because men make up most (i.e. about 80%) of the formal global workforce as compared to women (i.e. around 50%). In addition, the bias exists because of occupational segregation on a larger scale. The primary use of the WBGT is to set work safety limits and safety thresholds are misleading being set for men: For example, pregnant women are a group vulnerable to heat stress, and, when a woman has formal employment, most labour laws require women to work for at least part of their pregnancy (Bisello and Mascherini 2017) (Mutambudzi et al. 2011). Only three studies on WBGT and heat safety limits, however, included pregnant women in their samples, and two of these studies were conducted in The Gambia (Bonell et al. 2020, 2023). Research on women and pregnant women is needed to create labour laws that protect the whole workforce. Most countries have developed some form of policies to ensure equal opportunity and workers’ rights, as well as to improve working conditions, but few studies we evaluated examined aspects of social vulnerability and inequity, pre-existing medical conditions, or the defined vulnerable groups. This gap indicates that this should be an area of future research, enabling evidence to be gathered that can be used as a basis for policies that work for all members of society (Bisello and Mascherini 2017; Mutambudzi et al. 2011; Sevak et al. 2015). There is evidence that some countries have already adapted the thresholds of WBGT to their individual climates for example in Japan and Taiwan, this depends on how the threshold is being used, in Japan for heat early warning and Taiwan to reduce hospitalization in heatwaves (Cheng et al. 2019; Oka et al. 2023).

The examined text extracts from WBGT studies clustered around occupation but only broadly addressed some main outdoor occupations, such as farming, different types of sports and athletics, and forms of mining. To assess whether the current safety thresholds are appropriate across a range of occupations, other populations that would be useful to study in the future are aquaculture workers, waste refuse workers, and a variety of outdoor engineers (i.e. green energy workers), as well as emergency service providers, coastguards, and mountain rescue service providers (Vannuccini et al. 2018; Yang et al. 2018).

### WBGT: a metric with many approximations

Confusion around the WBGT metrics is apparent from the literature base, when reviewing the texts extracted from the 913 papers, many different WBGT approximations were identified, or parts were studied (i.e. globe temperature and wet bulb temperature) (*n* = 253) (Minard 1961). The wet bulb temperature serves as a metric for survivability levels, with the threshold for survival estimated to be approximately 35 °C of dry bulb temperature combined with 100% relative humidity. However, Vecellio et al. (2022) highlighted that the actual threshold is considerably lower than 35 °C. The difference between the WBGT and wet bulb temperature is the inclusion of incidence of solar radiation or globe temperature. All components of the WBGT, i.e. wet bulb temperature, globe temperature, and air temperature (dry bulb temperature), can be used as heat stress metrics in some way, but all have different uses and risk thresholds (Budd 2008).

The caveat to consider when using the WBGT is that the instruments used for operational meteorological measurement are expensive, and WBGT is often not observed (Brimicombe et al. 2023; Kong and Huber 2022). There is research on ways to resolve this (Krüger et al. 2023; Morawska et al. 2018; Salamone et al. 2022). However, the WBGT is the metric of metrics, and many approximations and ways to calculate the WBGT have emerged. The second most frequently cited paper on the WBGT uses approximation (Dunne et al. 2013). We also evaluated the use of another approximation in the literature: the environmental stress index (ESI) (Moran et al. 2001). Two additional approximations identified were the Swedish WBGT(Ljungberg et al. 1979) and the WBGT simple, which was developed by the American College of Sports Medicine (American College of Sports Medicine 1987). Sometimes, climate change projections do not consider radiation or approximate the challenges associated with longer-term radiation models (Dunne et al. 2013; Orlov et al. 2020). This is important because the warning thresholds and heat risk projections from studies that use an approximate WBGT can vary drastically in comparison to absolute WBGT values(Kong and Huber 2022; Simpson et al. 2023) and may lead to maladaptive practices. In addition, there is an uptake of climate change attribution studies being used in litigation cases, WBGT is an important metric in worker safety, making it a key heat stress metric that could be important in such cases (Marjanac and Patton 2018; Setzer and Higham 2021). As such it is even more important to be as accurate and robust as possible when considering legal systems.

The WBGT should be measured using standard equipment in real-world settings (Int Org Standard 2017). Weather forecasting and climate modelling should use the WBGT_Liljegren_ or WBGT_Brimicombe_, if possible (Brimicombe et al. 2023; Liljegren et al. 2008). More research is needed to assess the impact of applying radiation schemes from climate models to approximate and calculate heat and thermal comfort indices and error quantifications or to determine the accuracy of heat stress projections.

### The role of heat adaptation

Even though international standards are in effect, we noted high variance and differences in the WBGT thresholds considered in studies, with most of our understanding of thresholds coming from athletic studies. More standardisation is need across research studies to support the operational role WBGT plays in ensuring a safe working environment. More research is also needed to address health aspects (i.e. levels of hospitalisation) and to see if this threshold is comparable to heat safety thresholds of heat stress in the workplace. However, we did find that the median heat stress thresholds used in the identified studies for all available age and sex categories fell within 1.5 °C of the threshold for the resting metabolic rate for acclimatised or unacclimatised individuals (Int Org Standard 2017). In addition, studies examining the effects of using protective clothing defined higher safe work thresholds, but also demonstrated that safe work limits exist even workers wear such garments (Bishop et al. 1991; Montain et al. 1994). We also found studies that assessed other types of heat adaptation options, such as using natural ventilation for the indoor WBGT, misting fans, green roofs, and urban greening (Castiglia Feitosa and Wilkinson 2020; Meng et al. 2021; Wang et al. 2016). More studies need to be carried out to measure the effectiveness of using such tools, including studies to determine their cost, so that managers of workplaces and other settings become aware a range of suitable options that can be used to maintain safe WBGT values.

Heat adaptation can reduce the rising impact of heat on populations and vulnerable groups but can only go so far to limit impacts because the heat survivability limit has been breached for short periods in some regions (Vecellio et al. 2022). Some studies indicated whether we could achieve net zero carbon emissions and maintain a safe thermal environment in the workplace by installing air conditioning (Amada et al. 2022). Another study explored how much energy would be required to ensure that thermal comfort levels were met by employing different strategies, including air conditioning, as indicated by the indoor WBGT (Latha et al. 2020). Both climate adaptation and mitigation policies need to work together to reduce the impact of heat stress and maintain an environment within the survivability limits.

### Bridging the gap between heat exposure and vulnerability

We call for robust work regulations that ensure the thermal comfort of the workforce, our results demonstrate that the evidence-base already can be used to assist the development of safety thresholds for many sub-groups (such as young adults and adult women). We call for more research for socially vulnerable groups, by first carrying out pilot studies on existing regulations, researchers can focus on examples of social vulnerability and pre-existing medical conditions and examine the effects of sex and age in different climate zones.

Other policy and research gaps we identified indicate a strong need to evaluate heat safety limits for neonates, infants, children, and pregnant and postpartum women. To assess the effectiveness of strategies, evidence must be gathered and used to develop policies that ensure the overall safety of workforces and society in the future. For example, few studies have been performed to determine the effectiveness of using different heat adaptation tools, such as natural ventilation in indoor settings, misting fans, green roofs, and urban greening. Such studies would be beneficial, as they would give insight into when air conditioning use is acceptable and when it can be avoided, given its high energy demand.

In summary, our findings demonstrated that the evidence for current heat safety thresholds and policies indicate that these are not safe or healthy for all members of the working population and society. In addition, our results also show that climate zones, acclimatisation to heat, and cooling clothing can raise the safe limit to some extent, enabling people to work and live in certain areas, even where they are exposed to extreme heat. The results, however, also show that a maximum limit is still needed. Studies on vulnerable groups and those that measure safe heat levels are needed to help all members of the population adapt to heat stress effectively. Commitments to support the net zero transition must increase to avoid further breaches of the survivability limit.

## Electronic supplementary material

Below is the link to the electronic supplementary material.

Supplementary Material 1

Supplementary Material 2

## Data Availability

All data are freely available. Processed data are available as a supplementarydocument.

## Code availability

All coding analyses and visualisation took place in Python and are available upon request.
